# Microbe Profile: *Thermococcus kodakarensis*: the model hyperthermophilic archaeon

**DOI:** 10.1099/mic.0.000839

**Published:** 2019-08-22

**Authors:** Haruyuki Atomi, John Reeve

**Affiliations:** ^1^​ Department of Synthetic Chemistry and Biological Chemistry, Graduate School of Engineering, Kyoto University, Katsura, Nishikyo-ku, 615-8510 Kyoto, Japan; ^2^​ Department of Microbiology, Ohio State University, Columbus, OH 43210, USA

**Keywords:** archaea, *Thermococcus kodakarensis*, hyperthermophile, genetics

## Abstract

*
Thermococcus kodakarensis* is a hyperthermophilic *Euryarchaeon* that grows well under laboratory conditions and, being naturally competent for genetic transformation, it has become a widely studied experimental model species. With the genome sequence available since 2004, combining genetic, enzymological and structural biochemical approaches has revealed previously unknown and unanticipated features of archaeal molecular biology and metabolism. *T. kodakarensis* DNA polymerase is already commercialized and with the details of metabolism and hydrogenase available, generating H_2_ from biopolymers solubilized at high temperatures, most notably chitin, now seems a very attractive possibility as a renewable energy bioprocess.

## Taxonomy

Domain: *Archaea*; phylum: *Euryarchaeota *; class: *Thermococci *; order: *Thermococcales *; family: *Thermococcaceae *; genus: *Thermococcus *; species: *T. kodakarensis *.

## Properties

*T. kodakarensis* is an obligately anaerobic, hyperthermophilic heterotrophic marine *Archaeon*. Growth under laboratory conditions occurs from 60 to 100 °C, from pH 5 to 9 and with 1 to 5 % NaCl, optimally at 85 °C, pH 6.5 and 3 % NaCl. The cells are polyploid (7–19 genomes/cell), motile, irregular cocci, ~1.5 µm in diameter, surrounded by a cell membrane with diphytanylglycerol di- and tetra-ether lipids and an outer glycoprotein S-layer. Motility is driven by a lophotrichous bundle of 4–14 archaella, functional analogues of bacterial flagella but evolutionarily related to bacterial type IV pili and type II secretion systems. Heterotrophic growth occurs with generation times <1 h in media containing yeast extract, tryptone, peptone and free amino acids with sulfur (S^0^) as the terminal electron acceptor, generating H_2_S. Growth also occurs in the absence of S^0^ by fermentation of starch, malto-oligosaccharides, cyclodextrins or pyruvate generating H_2_.

## Genome

The *T. kodakarensis* genome sequence was first reported in 2005 and again in 2017, revealing 35 single-nucleotide changes that had occurred in the single circular 2 088 737 bp genome during the intervening years [1]. The genome annotation predicts 2306 encoded proteins, with the functions of >200 already experimentally investigated. There is one 16S rRNA-tRNA^Ala^-23S rRNA operon, two 5S rRNA genes, one 7S rRNA gene, 46 tRNA genes with introns in the tRNA^Met^ and tRNA^Trp^ genes, 4 prophage-related regions, 7 transposases associated with IS elements, 3 CRISPR loci, 9 putative restriction endonucleases and 49 type II toxin–antitoxin partnerships. As isolated, *T. kodakarensis* has no plasmids, but plasmids from other *Thermococcales* do replicate stably and provide the basis for extra-chromosomal expression systems when transformed into *T. kodakarensis *.

### Phylogeny

The *Thermococcales* are a distinct and cohesive branch within the *Euryarchaeota *, with three genera, *Thermococcus, Pyrococcus* and *Palaeococcus. T. kodakarensis* is 1 of 13 thermococcal species, distinguished by genome sequencing, and was isolated from a solfatara (102 °C, pH 5.8) on the beach of Kodakara Island, Japan and described in 1994 as *Pyrococcus* sp. KOD1. In 2004, it was reclassified as *T. kodakaraensis* strain KOD1 [2] and is now simply designated *T. kodakarensis *. Many *T. kodakarensis* strains have since been constructed and given different alpha-numeric designations, but they are all derivatives of the KOD1 isolate.

## Key features and discoveries

*T. kodakarensis* was initially attractive for research because of its metabolic flexibility and ease of laboratory culture, but it was the report of natural competence for genetic transformation in 2003 [3] that propelled it into the role of model archaeon. Laboratory procedures have since been developed to precisely delete or add genes to the *T. kodakarensis* genome, statistically identify essential genes and randomly mutagenize the genome. Temperature-sensitive mutants that grow at 85 °C but not at 95 °C, a protein secretion system and a fluoride-dependent riboswitch that regulates the expression of cloned genes are also available.

Combining genetics and biochemistry has revealed novel and unanticipated features of archaeal molecular biology and metabolism. The essential (or not) status of many proteins (PCNAs, MCMs, cdc6, recJ/cdc45, GAN, Fen1, RNase H1, RadA, RadB, TFBs) bioinformatically identified as potential components of the DNA replication and transcription machineries has been established. Genome replication has been shown to be dependent on DNA polymerase D (polD) rather than polB and, surprisingly, not to require cdc6 or an origin of replication [1]. Either of the two archaeal histones is sufficient for viability and the *in vivo* structure of chromatin has been visualized by atomic force microscopy. Coupling of transcription and translation, the crystal structure of RNA polymerase and the roles of transcription factors in gene expression and overcoming archaeal nucleosome barriers have been established. Global genome expression has been documented in transcriptome and proteome studies.

Investigation of the absence of enzymes that are essential for metabolism in other species in the genome annotation led to the discovery of novel pathways. *T. kodakarensis* lacks the uptake systems and so does not grow on glucose and maltose, but secretes enzymes that depolymerize starch and chitin, generating hexose oligomers that are transported and catabolized for growth. Glycolysis by a modified Embden–Meyerhof pathway employs both a conventional pyruvate kinase and a phosphoenolpyruvate (PEP) synthase to convert PEP to pyruvate. Glyceraldehyde 3-phosphate (GAP) conversion to 3-phosphoglycerate is catalyzed by a GAP : ferredoxin oxidoreductase and a non-phosphorylating GAP dehydrogenase. A phosphorylating GAP dehydrogenase and phosphoglycerate kinase participate exclusively in gluconeogenesis, together with a structurally novel protein that has both fructose-1,6-bisphosphatase and fructose-1,6-bisphosphate aldolase activity. A separate, atypical fructose-1,6-bisphosphate aldolase functions in glycolysis. The classical pentose phosphate pathway is absent and, in its place, a ribulose monophosphate pathway provides pentoses. A pentose bisphosphate pathway has been discovered that degrades and recycles the pentose moieties from nucleosides and nucleotides [4]. Enzymes involved in the biosynthesis of cysteine, serine, tryptophan, lysine and proline, and ADP-forming acyl-CoA synthetases required for amino acid catabolism have been characterized. The biosynthetic pathways to coenzyme A, long and branched-chain polyamines and salvage pathways for NAD^+^ have been established.

The electrons from catabolism are used primarily to reduce NADP^+^ or ferredoxins [5] that are re-oxidized by generating H_2_, or H_2_S when S^0^ is present. A membrane-bound Ni–Fe hydrogenase couples H_2_ production to the generation of a proton gradient that is converted into a Na^+^ gradient that drives ATP synthesis. Crystal structures and action mechanisms have been established for all of the Hyp accessory proteins and HycI proteases that construct the catalytic center of the Ni–Fe hydrogenase, alone and in complexes with each other or with the hydrogenase subunits [6]. Driven by both inherent interest and the commercial interest in hyperthermophilic enzymes, 150 high-resolution structures of *T. kodakarensis* proteins are already in the Protein Data Bank.

*T. kodakarensis* has multiple systems that assist in protein folding and structural maintenance, including members of both the HSP60 and CpkB families of chaperonins, PfdA/PfdB and PfdC/PfdD prefoldins, and CpkA and CpkB small heat shock proteins. PfdC/PfdD and CpkB are synthesized in response to heat shock, whereas PfdA/PfdB is constitutively expressed and CpkA synthesis is activated by cold shock, possibly facilitating the growth of *T. kodakarensis* at low (60 °C) temperatures [7].

### Open questions

- What is the mechanism of origin- and cdc6-independent genome replication? Does this mechanism involve recombination and does that play a role in the atypical natural genetic competence of *T. kodakarensis *? Why were the origin-like sequence and cdc6 retained by the wild-type strain?
- How does a *T. kodakarensis* cell accommodate, duplicate and segregate 10–20 genomes each cell cycle?
- What mechanisms regulate the novel metabolic pathways and how are metabolic intermediates and cofactors protected from thermal degradation in *T. kodakarensis *?
- How does *T. kodakarensis* cope with or utilize the oxygenase activity of RuBisCO?
- Why does *T. kodakarensis* grow relatively slowly on chitin? Is H_2_ generation from chitin a commercially practical renewable energy bioprocess [8]?
